# Botulinum Toxin B Affects Neuropathic Pain but Not Functional Recovery after Peripheral Nerve Injury in a Mouse Model

**DOI:** 10.3390/toxins10030128

**Published:** 2018-03-18

**Authors:** Alba Finocchiaro, Sara Marinelli, Federica De Angelis, Valentina Vacca, Siro Luvisetto, Flaminia Pavone

**Affiliations:** 1 National Research Council of Italy-CNR, Institute of Cell Biology and Neurobiology-IBCN, 00143 Roma, Italy; alba.finocchiaro@hotmail.it (A.F.); sara.marinelli@cnr.it (S.M.); valentina.vacca@ibcn.cnr.it (V.V.); 2 Department of Psycology, PhD School of Behavioural Neuroscience, Sapienza University, 00185 Roma, Italy; 3 IRCCS Santa Lucia Foundation, 00143-Roma, Italy; federica.deangelis@uniroma1.it

**Keywords:** nerve regeneration, Schwann cells, glia, spinal cord, immunohistochemistry, allodynia, weight bearing, sciatic static index, walking track analysis

## Abstract

Clinical use of neurotoxins from *Clostridium botulinum* is well established and is continuously expanding, including in treatment of pain conditions. *Background*: The serotype A (BoNT/A) has been widely investigated, and current data demonstrate that it induces analgesia and modulates nociceptive processing initiated by inflammation or nerve injury. Given that data concerning the serotype B (BoNT/B) are limited, the aim of the present study was to verify if also BoNT/B is able not only to counteract neuropathic pain, but also to interfere with inflammatory and regenerative processes associated with the nerve injury. *Methods*: As model of neuropathic pain, chronic constriction injury (CCI) of the sciatic nerve was performed in CD1 male mice. Mice were intraplantarly injected with saline (control) or BoNT/B (5 or 7.5 pg/mouse) into the injured hindpaw. For comparison, another mouse group was injected with BoNT/A (15 pg/mouse). Mechanical allodynia and functional recovery of the injured paw was followed for 101 days. Spinal cords and sciatic nerves were collected at day 7 for immunohistochemistry. *Results and Conclusions:* The results of this study show that BoNT/B is a powerful biological molecule that, similarly to BoNT/A, can reduce neuropathic pain over a long period of time. However, the analgesic effects are not associated with an improvement in functional recovery, clearly highlighting an important difference between the two serotypes for the treatment of this chronic pain state.

## 1. Introduction

The interest in botulinum neurotoxins (BoNTs) has been growing in the last years because they have become important therapeutic agents for many pathological conditions, including pain [1,2,3,4]. This was allowed by better comprehension of the mechanism of action of BoNTs [5,6]. 

Seven BoNT serotypes (A–G) have been recognized up until now, with a growing number of subtypes continuously identified [7]. All BoNTs are zinc endopeptidases that selectively cleave one of the SNARE (soluble *N*-ethylmaleimide-sensitive factor attachment protein receptor) proteins essential for the formation of SNARE complex, the core component of the eukaryotic neuroexocytosis [8]. Owing to their zinc endopeptidase activity, BoNT/A and /E cleave SNAP25 (synaptosomal-associated protein of 25 kDa), BoNT/B, /D, /F, and /G cleave VAMP (vesicle-associated membrane protein), while BoNT/C cleaves both SNAP25 and syntaxin [9]. 

Most of preclinical data on the analgesic effects of BoNTs derive from experiments with BoNT/A, which is also the serotype with the long-lasting action duration and lower toxicity. In the past years, we demonstrated the antiallodynic efficacy of BoNT/A in the mouse model of chronic constriction injury (CCI) of the sciatic nerve [10,11,12,13,14]. Antiallodynia was accompanied by structural changes in injured nerve and improvements in functional recovery of the injured hindlimb. In details, a single intraplantar (ipl) injection of BoNT/A in the injured hindpaw was sufficient to (i) inhibit glial cell activation; (ii) modulate pro- and anti-nociceptive interleukins; (iii) accelerate processes related to sciatic nerve regeneration; and (iv) improve functional recovery from CCI-induced atrophy of the injured hindpaw. 

From a clinical view, BoNT/A has been approved by FDA for treatment of a wide variety of clinical conditions [2,15], but a downside may limit its use. As a matter of fact, the action of BoNTs is reversible, and after repeated treatment, BoNT/A can induce the phenomenon of tolerance, generating the need to continue therapy using other serotypes. Nowadays, the only serotype commercially available in replacement of BoNT/A is BoNT/B, but in view of the relevant clinical differences of the two serotypes [16], BoNT/B has been approved only for cervical dystonia.

As for basic science, only few studies focused on the use of BoNT/B in chronic pain. Peripheral administration of BoNT/B reduced peripheral inflammation and spinal nociceptive processing in inflammatory pain models, such as carrageenan [17], capsaicin [18], and formalin [18,19], and yields a long-lasting attenuation of allodynia in mice subjected either to spinal nerve ligation [20,21] or to cisplatin administration [21]. 

As suggested by the commentary of Pavone and Ueda [22], it is extremely important to understand the mechanisms underlying the action of BoNT serotypes other than serotype A. We decided to verify not only if BoNT/B could counteract CCI-induced neuropathic pain for a long time interval, but also if it could affect, as occurs for BoNT/A, the regenerative processes of the peripheral injured nerve, as well as the functional recovery of the injured hindlimb. In addition, we performed, in BoNT/B-treated CCI mice, immunohistochemistry analysis of spinal cord, looking for the expression of markers of activation of glial cells. 

## 2. Results

### 2.1. Effects of BoNT/B on Mechanical Nociceptive Threshold in CCI Mice

**Figure 1:**
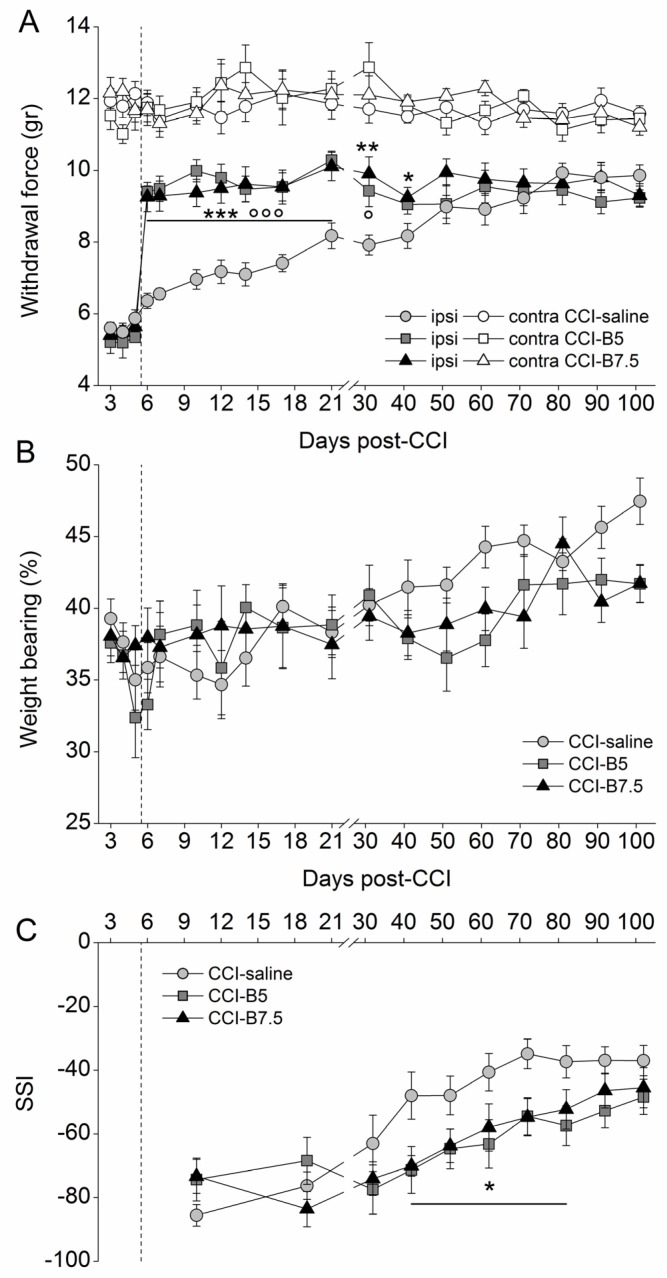
Mechanical allodynia and functional recovery in chronic constriction injury (CCI)-mice. (**A**) Mechanical allodynia measured on ipsilateral (closed symbols) and contralateral (open symbols) hindpaw of CCI-mice after a single ipsilateral injection of saline (CCI-saline: ○,●), BoNT/B 5 pg/paw (CCI-B5: □,■) and BoNT/B 7.5 pg/paw (CCI-B7.5: △,▲). Dotted line indicates the day of injection. In CCI-BoNT/B mice groups the strong antiallodynic effect was observed starting from the day after the injection and persisted for a very long time. Statistical symbols: (ooo) *p* < 0.001, (o) *p* < 0.05 for CCI-B5 vs. CCI-saline; (***) *p* < 0.001, (**) *p* < 0.01, (*) *p* < 0.05 for CCI-B7.5 vs. CCI-saline. (**B**) Weight bearing calculated as ratio of the weight distribution between the hindpaws, in CCI-saline (●), CCI-B5 (■) and CCI-B7.5 (▲) mice. In all mice groups there was a slow but incomplete functional recovery over time. The CCI-BoNT/B mice did not show difference compared to CCI-saline mice. (**C**) Walking track analysis: sciatic static index, SSI, calculated from hindpaws’ footprints in CCI-saline (●), CCI-B5 (■) and CCI-B7.5 (▲) mice. BoNT/B slowed down the functional recovery of mice: CCI-BoNT/B mice expressed more negative values in respect to the CCI-saline mice. Statistical symbols: (*) *p* < 0.05 both for CCI-B5 and CCI-B7.5 vs. CCI-saline.

To verify the analgesic properties of BoNT/B, we analyzed its effect on mechanical nociceptive threshold in CCI mice (Figure 1A). In CCI-saline mice, the mechanical nociceptive threshold in the ipsilateral hindpaw was around 50% lower compared to contralateral hindpaw. Since mice withdrew their ipsilateral hindpaw after very low stimuli (5–6 g), which did not evoke any reaction in contralateral hindpaw, we considered this response as allodynia. The ipsilateral allodynia was maintained for at least 3 weeks and, even if reduced, was still present after 3 months. On the contrary, a single ipl injection of BoNT/B (5 or 7.5 pg/paw at D5) in CCI mice (CCI-B5 and CCI-B7.5) clearly antagonizes allodynia, as indicated by the clear-cut enhancement of the ipsilateral withdrawal threshold, at values corresponding to 80% of the contralateral withdrawal threshold. Both in CCI-B5 and CCI-B7.5 mice, the antiallodynic effect was observed already at D6 (the day after the injection) and the significant difference with CCI-saline mice persisted for a very long time. Two-way ANOVA for repeated measures carried out for ipsilateral allodynia showed a significant main effect for treatment (F_2,27_ = 10.941; *p* < 0.0003), days (F_17,459_ = 68.739; *p* < 0.0001) and treatment x days interaction (F_34,459_ = 7.031; *p* < 0.0001). Post hoc comparisons vs. CCI-saline mice confirmed a significant difference (Tukey–Kramer, *p* < 0.05) from D6 to D31 for CCI-B5, and from D6 to D41 for CCI-B7.5 mice.

### 2.2. Effects of BoNT/B on Functional Recovery after CCI

Functional recovery was examined by (i) measuring the weight bearing between the two hindlimbs (incapacitance test); and, (ii) walking track analysis (footprint test), followed by calculation of the sciatic static index (SSI).

Figure 1B shows the reduction of the weight bearing to value <50% in CCI-mice, indicating a displacement of weight distribution toward the contralateral hindlimb. The graph indicates that, independently from the treatment, there was a slow recovery over time. Two-way ANOVA for repeated measures showed not significant effects for treatment (F_2,27_ = 1.013, *p* = 0.3765), days (F_17,459_ = 5.341, *p* < 0.0001), and treatment x days interactions (F_34,459_ = 1.226, *p* = 0.1824).

Figure 1C shows the SSI for CCI-saline, CCI-B5, and CCI-B7.5 mice calculated with the walking track analysis. All CCI mice manifested a severe impairment of motor function, as revealed by SSI values (around 80) until D32; then they showed a slow, but incomplete, functional recovery. In particular, in CCI-saline mice, the SSI values were slowly increased during the overall time course but, 2 months after CCI, impairments in functional recovery were still observed. Both CCI-B5 and CCI-B7.5 mice did not show improvement, indeed, their SSI values worsened in comparison to CCI-saline mice. At D101, CCI-B5 and CCI-B7.5 mice expressed SSI values similar to those of CCI-saline mice. Two-way ANOVA for repeated measures showed a significant main effect for days (F_9,243_ = 32.786; *p* < 0.0001) and for treatment x days (F_9,243_ = 2.867; *p* = 0.0001). Post hoc comparisons vs. CCI-saline showed a significant difference (*p* < 0.05) from D42 to D82, for both CCI-B5 and CCI-B7.5 mice.

### 2.3. Effect of BoNTs on Cytoskeleton and Myelin Sheath of Injured Sciatic Nerve

**Figure 2:**
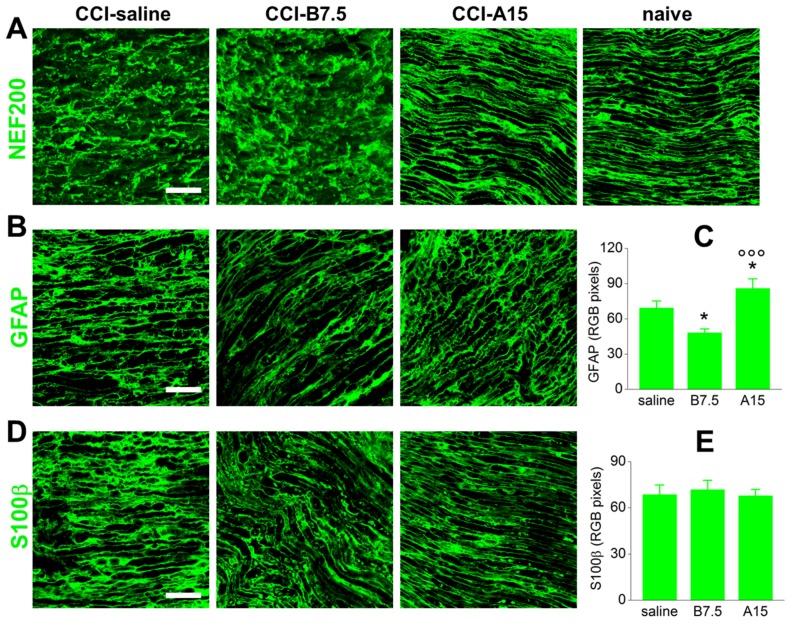
IF analysis of NF200, GFAP, and S100β in injured sciatic nerve. (**A**) Representative examples of confocal images at high magnification (63×) showing IF expression of NF200 in sciatic nerve sections, proximal to the lesion, from CCI-saline, CCI-B7.5, and CCI-A15 compared to naïve mice. As evidenced by morphological staining with NF200, CCI-saline and CCI-B7.5 showed similar tissue morphology with disjoined fibers, while CCI-A15 was similar to naïve mice, with almost regular tissue morphology and uniform and compact structure. Scale bar: 50 μm. (**B**) Representative examples of confocal images at high magnification (63×) showing IF expression of GFAP (green) and its quantification by RGB analysis (**C**), in sciatic nerve sections from CCI-saline, CCI-B7.5, and CCI-A15 mice. Scale bar: 50 μm. RGB analysis reveals an increased expression of GFAP in CCI-A15 mice and a decreased expression of GFAP in CCI-B7.5 mice, with respect to CCI-saline. (**D**) Representative examples of confocal images at high magnification (63×) showing IF expression of S100β (green) and its quantification by RGB analysis (**E**), in sciatic nerve sections from CCI-saline, CCI-B7.5, and CCI-A15 mice. Scale bar: 50 μm. RGB analysis reveals no different expression of S100β in all CCI mice groups. Statistical symbols: (*) *p* < 0.05 vs. CCI-saline; (ooo) *p* < 0.001 vs. CCI-B7.5.

The axonal injury caused by CCI induces structural changes in damaged nerves, which begin almost immediately and determine a progressive degradation of cytoskeletal proteins. Ipsilateral sections of sciatic nerves were incubated with rabbit polyclonal antibody anti-neurofilament-200 (NF200), a marker of large-diameter axons. As evidenced by representative examples in Figure 2A, both CCI-saline and CCI-B7.5 mice showed disjointed and broken up tissue, with loss of axonal integrity and neurofilaments not equally distributed along the fibers. Conversely, in CCI-A15 mice, neurofilaments appeared almost uniform, with a tissue presenting a compact regular structure, more similar to naïve mice than to saline CCI mice. 

Figure 2B shows IF of GFAP in ipsilateral sciatic nerve sections taken from CCI-saline, CCI-B7.5, and CCI-A15 mice. GFAP is a cytoskeleton constituent of Schwann cells (SCs) expressed both by immature dedifferentiate and mature non-myelinating cells. After peripheral nerve injury, SCs lose contact with axons, increase their GFAP expression, and acquire an immature dedifferentiated phenotype resembling non-myelin forming SCs. As previously reported [11], the expression of GFAP in injured sciatic nerves of all CCI mice groups was evaluated by means of RGB pixel analysis of IF images (Figure 2C). One-way ANOVA evidenced a significantly (F_2,38_ = 11.913; *p* < 0.0001) different expression of GFAP among groups. In respect to CCI-saline mice, post hoc comparisons showed that GFAP expression was significantly increased in CCI-A15 mice (Fisher’s PLSD analysis, *p* = 0.0274) and significantly reduced in CCI-B7.5 mice (*p* = 0.0397), respectively. The expression of GFAP was also significantly different (*p* < 0.0001) between CCI-B7.5 and CCI-A15 mice. 

Figure 2D shows IF of S100β marker of SCs, in ipsilateral sciatic nerve sections taken from CCI-saline, CCI-B7.5, and CCI-A15 mice. S100β is a glial-specific protein localized in the cytoplasm and nucleus of a wide range of cells, derived from neural crest, including SCs. In SCs, S100β is expressed both by immature dedifferentiating and mature myelinating cells. Differently from the expression of GFAP, the expression of S100β was not significantly different among the different CCI mice groups (quantification in Figure 2E).

### 2.4. Effects of BoNTs on Myelin-Associated Protein Expressed by SCs

Schwann cells play a key role in the clearance of myelin debris from damaged nerve. The loss of axon–SC contact is a signal that causes SC proliferation, an important event that promotes axon regeneration. During proliferation, myelinating and non-myelinating SCs change their phenotype from differentiated to dedifferentiated cells, and this switch is associated with expression of regeneration- and myelin-associated proteins (e.g., P0, PMP22). 

To avoid enhancement of pro-inflammatory status associated with myelin debris and aggregates, SCs begin demyelination with fragmentation of the myelin sheath into small ovoid-like structures that occur near the Schmidt–Lanterman incisures [23,24].

**Figure 3:**
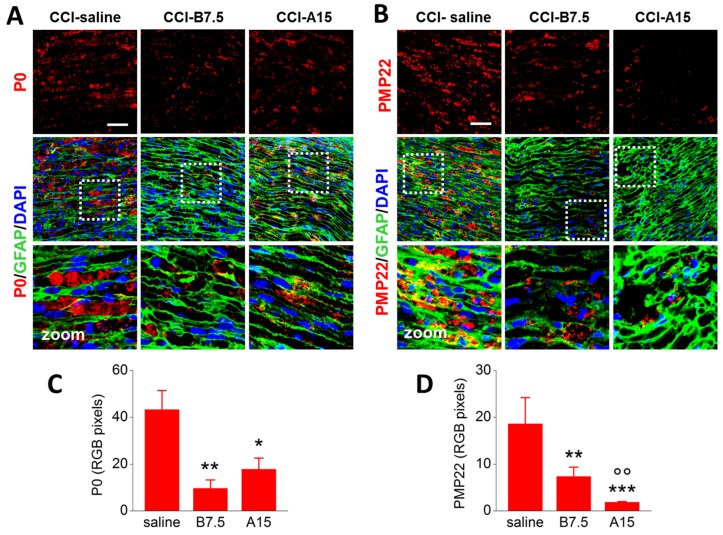
IF analysis of P0 and PMP22 in injured sciatic nerve. (**A**,**B**) Representative examples of confocal images at high magnification (63×) showing single IF expression of P0 or PMP22 (red), or double IF with GFAP (green), and their quantification by RGB analysis (**C**,**D**), in sciatic nerve sections from CCI-saline, CCI-B7.5, and CCI-A15 mice. White dotted squares in panels with double IF indicate the zone of the section where zooming (×2) was taken. Zoomed images: typical examples of myelin ovoids, which are more expressed in CCI-saline mice. Scale bar: 50 μm. RGB analyses reveal lower expression of P0 and PMP22 in CCI-A15 and CCI-B7.5 with respect to CCI-saline mice. Statistical symbols: (*) *p* < 0.05, (**) *p* < 0.01 vs. CCI-saline; (oo) *p* < 0.01 vs. CCI-B7.5.

Figure 3 shows IF for either P0 (marker of a major peripheral myelin protein; Figure 3A) or PMP22 (marker of peripheral myelin protein 22; Figure 3B), alone or co-stained with GFAP for SCs (green) and DAPI for nuclei (blue) in ipsilateral sciatic nerve sections taken from CCI-saline, CCI-B7.5, and CCI-A15 mice. As evidenced by the zoomed images in Figure 3A,B, both P0 and PMP22 were found aggregated and accumulated in characteristic ovoids in CCI-saline mice. Different results were observed in samples from CCI-B7.5 and CCI-A15 mice, where the presence of myelin aggregates in ovoids was less evident, and staining of P0 and PMP22 was reduced in respect to CCI-saline. 

RGB analysis and one-way ANOVA confirmed the different expression of P0 (Figure 3C) and PMP22 (Figure 3D) in the different CCI mice groups. As for P0, ANOVA indicated a significant main effect for treatment (F_2,17_ = 5.468; *p* = 0.0147); post hoc comparisons showed significantly reduced expression of P0 in both CCI-B7.5 (*p* = 0.0052) and CCI-A15 (*p* = 0.0197) vs. CCI-saline mice. Instead, CCI-B7.5 and CCI-A15 did not show any differences between them. As for PMP22, ANOVA indicated a significant main effect for treatment (F_2,18_ = 7.952; *p* = 0.0034); post hoc comparisons showed a significant reduced expression of PMP22 in both CCI-B7.5 (*p* = 0.0019) and CCI-A15 (*p* = 0.0009) vs. CCI-saline, and, differently from P0, in CCI-A15 vs. CCI-B7.5 (*p* = 0.0029). 

### 2.5. Effect of BoNTs on the Immune Cells after CCI

After peripheral nerve damage, resident mast cells are the first immune cells to be activated. Activated mast cells degranulate and release a variety of proinflammatory mediators, which contribute to recruitment of neutrophils and, as a consequence, of infiltrating activated hematogenous macrophages [25], facilitating a rapid elimination of myelin debris and nerve regeneration. 

**Figure 4:**
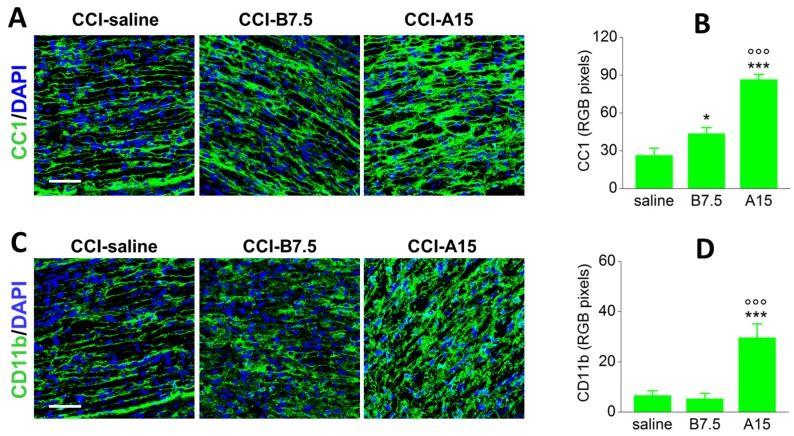
IF analysis of CC1 and CD11b in injured sciatic nerve. (**A**) Representative examples of confocal images at high magnification (63×) showing IF expression of CC1 (green) and its quantification by RGB analysis (**B**) in sciatic nerve sections from CCI-saline, CCI-B7.5, and CCI-A15 mice. Scale bar: 50 μm. RGB evaluation reveals an increased expression of CC1 in CCI-B7.5 and CCI-A15 mice compared to CCI-saline mice. This increase is particularly evident in CCI-A15 mice. (**C**) Representative examples of confocal images at high magnification (63×) showing IF expression of CD11b (green) and its quantification by RGB analysis (**D**) in sciatic nerve sections from CCI-saline, CCI-B7.5 and CCI-A15 mice. Scale bar: 50 μm. RGB evaluation reveals an increased expression of CD11b only in CCI-A15 mice compared to CCI-saline mice. Statistical symbols: (***) *p* < 0.001 vs. CCI-saline; (ooo) *p* < 0.0001 vs. CCI-B7.5.

Figure 4A shows the IF of CC1 (green), marker of chymase 1, a protein expressed by activated mast cells, in sciatic nerve sections from CCI mice. Compared to saline mice, the expression of mast cells was enhanced in both CCI-B7.5 and CCI-A15 mice groups. RGB analysis and one-way ANOVA (Figure 4B) for CC1 indicated a significant main effect for treatment (F_2,24_ = 38.224; *p* < 0.0001). Post hoc comparisons confirmed a significant difference of both CCI-B7.5 (Fisher’s PLSD analysis; *p* = 0.0222) and CCI-A15 (*p* < 0.0001) vs. CCI-saline mice, and of CCI-B7.5 vs. CCI-A15 mice (*p* < 0.0001).

Figure 4C shows the IF of CD11b (green), marker of peripheral macrophages, in the sciatic nerves from CCI mice. Compared to CCI-saline mice, the expression of macrophages was highly enhanced in CCI-A15 mice, but not in CCI-B7.5 mice group. RGB analysis and one-way ANOVA (Figure 4D) for CD11b expression indicated a significant main effect for treatment (F_2,24_ = 14.072; *p* < 0.0001); post hoc comparisons confirmed a significant difference of CCI-A15 vs. CCI-saline (*p* = 0.0002) and CCI-B7.5 (*p* < 0.0001). 

### 2.6. Effect of BoNT/B on Spinal Cord after CCI

After nerve injury, activation of glial cells, both microglia and astrocytes, is observed in spinal cord. Microglia activation, characterized by phosphorylation of p38 MAP kinase (p-p38), induces the production and secretion of proinflammatory cytokines that contribute to the onset and maintenance of pain hypersensitivity. 

**Figure 5:**
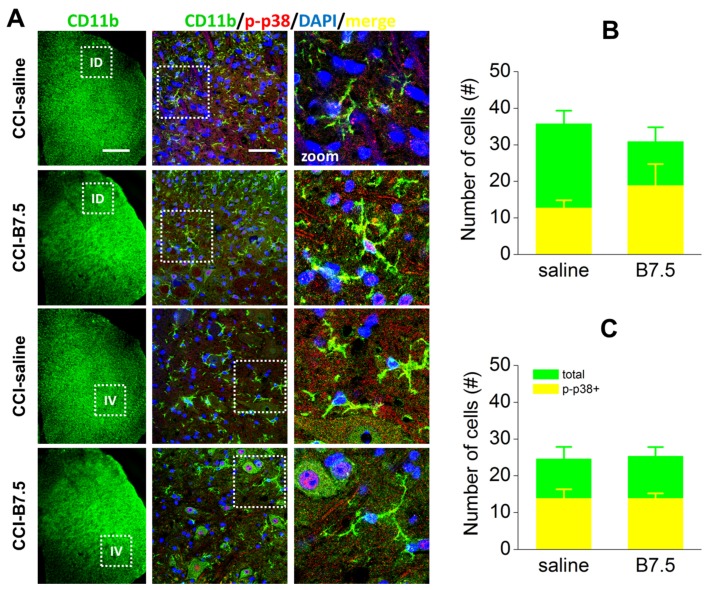
IF analysis of CD11b and p-p38 colocalization in ipsilateral spinal horns. (**A**) Examples of confocal images at 10× or 63× magnification showing double IF expression of CD11b (green), p-p38 (red), and CD11b/p-p38 (yellow) cells in ipsilateral dorsal (ID) and ventral (IV) horns of spinal cord sections in CCI-saline and CCI-B7.5 mice. White boxes in 10× images indicate the zone of the spinal cord sections considered for the 63× images. White boxes in 63× images indicate the zone of dorsal or ventral horns considered for further zoomed (2×) images. Scale bars: 300 μm for 10×; 50 μm for 63×. Analysis of number of cells in ipsilateral dorsal (**B**) and ventral (**C**) horns indicates no difference between CCI-saline and CCI-B7.5 mice in the counting of total CD11b and CD11b/p-p38 IR cells.

To detect spinal microglia, we incubated transverse sections of L4/L5 spinal cord segment of CCI-saline and CCI-B7.5 mice with rat monoclonal antibody anti-CD11b, as a marker of microglia, and with rabbit polyclonal antibody anti-p-p38. Figure 5A shows the expression of microglia (CD11b; green), and their colocalization with p-p38 (red) in ipsilateral dorsal (ID) and ipsilateral ventral (IV) horns of CCI-saline and CCI-B7.5 mice. Figure 5B,C show the counting of total number of CD11b immunoresponsive (IR) cells and the number of CD11b IR cells colocalized with p-p38 (p-p38/CD11b) in ID and IV horns, respectively. One-way ANOVA for the expression of total CD11b and p-p38/CD11b IR cells indicated no significant difference between CCI-saline and CCI-B7.5 mice, both at ID horn (total CD11b: F_1,10_ = 0.804, *p* = 0.3911; p-p38/CD11b: F_1,10_ = 3.586, *p* = 0.0875) and IV horn (total CD11b: F_1,10_ = 0.028, *p* = 0.8716; p-p38/CD11b: F_1,10_ = 0.000).

**Figure 6:**
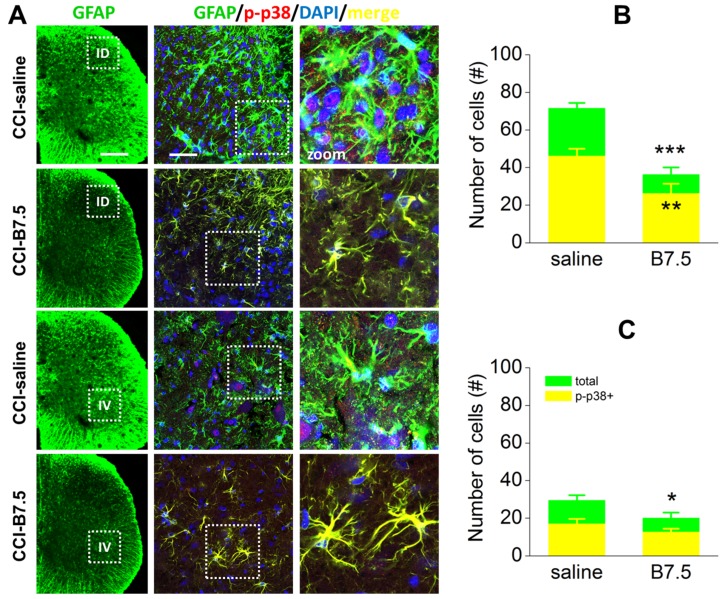
IF analysis of GFAP and p-p38 colocalization in ipsilateral spinal horns. (**A**) Examples of confocal images at 10× or high 63× magnification, showing double IF expression of GFAP (green), p-p38 (red) and GFAP/p-p38 (yellow) cells in ipsilateral dorsal (ID) and ventral (IV) horns of spinal cord sections in CCI-saline and CCI-B7.5 mice. White boxes in 10× images indicate the zone of the spinal cord sections considered for the 63× images. White boxes in 63× images indicate the zone of dorsal or ventral horns considered for further zoom (2×) images. Scale bars: 300 μm for 10×; 50 μm for 63×. Counting of total GFAP and GFAP/p-p38 IR cells in 63X dorsal (**B**) and ventral (**C**) horn images indicates a strong significant difference between CCI-saline and CCI-B7.5 for both total GFAP and GFAP/p-p38 IR cells in dorsal horns, and only for total GFAP IR cells in ventral horns. Statistical symbols: (***) *p* < 0.001, (**) *p* < 0.01, (*) *p* < 0.05 vs. CCI-saline.

Together with microglia, astrocyte activation also plays a role in chronic pain sensitization. After damage to peripheral nerves hypertrophy and proliferation of spinal astrocytes, which are accompanied by enhanced expression of GFAP, are observed. Figure 6A shows the IF of astrocytes (GFAP; green), and their colocalization with p-p38 (red) in ID and IV horns of CCI-saline and CCI-B7.5 mice. One-way ANOVA for the expression of total GFAP and p-p38/GFAP IR cells in ID horns (Figure 6B) indicated a strong significant decrease for both total GFAP (F_1,10_ = 59.387, *p* < 0.001) and p-p38/GFAP (F_1,10_ = 22.510, *p* = 0.0008) IR cells in CCI-B7.5 compared to CCI-saline group. Unlike ID horns, in IV horns (Figure 6C) one-way ANOVA for the expression of total GFAP and p-p38/GFAP IR cells showed a significant decrease only for total GFAP (F_1,10_ = 5.604, *p* = 0.0395), but not for the activated p-p38/GFAP (F_1,10_ = 2.169, *p* = 0.1715) IR cells. 

## 3. Discussion

The most considerable finding of this report is the remarkable difference between the two botulinum serotypes A and B in counteracting symptoms induced by CCI neuropathy: BoNT/B, differing from BoNT/A, reduced pain sensitivity in neuropathic mice without inducing any positive effect on functional recovery. This result is particularly relevant for its clinical implications, considering that both serotypes can be alternatively used when one of them resulted in the development of immunoresistance.

A single ipl injection of BoNT/B (5 or 7.5 pg/paw) exerted an almost immediate antiallodynic effect, which started from the day after injection and lasted for at least 30 days. The long-lasting analgesic effect resulted independently from the dose considered, the maximal effect being already observed at the lower dose of 5 pg/paw. This finding is compatible with the action of BoNTs as proteolytic enzymes, which is not directly proportional to the dose administered. 

In the past years, analgesic effects mainly of BoNT/A, but also of BoNT/B, have been reported in several pain models [3,4,17,26]. Interestingly, it was also demonstrated that BoNT/A, in addition to its analgesic effects, is able to accelerate functional rehabilitation in CCI-mice [11]. On the contrary, the present results show that BoNT/B not only did not improve but also slowed down functional recovery of mice, as shown by significant differences between CCI-BoNT/B and CCI-saline mice in walking track analysis. 

Differences in functional recovery between the two BoNTs serotypes may depend on different modulation by BoNTs of the processes favorable, or unfavorable, to the nerve regeneration. Table 1 shows a summary of changes in the expression of protein markers, associated to peripheral nerve injury, in the different experimental groups. 

After peripheral nerve injury, resident mast cells are found along the axons and juxtaposed to free nerve endings; they are activated from bacteria, viruses, and neuropeptides, including substance P, NGF, TNF-α, IL-1β, and many others. Activated mast cells degranulate and release a variety of proinflammatory mediators, including histamine, leukotrienes, and chemokines, which contribute to recruitment of neutrophils. Neutrophils, in turn, release several substances that help recruitment of activated hematogenous macrophages to the site of injury into degenerating nerve [25]; supplementation of resident macrophages by infiltrating activated hematogenous macrophages leads to a rapid elimination of myelin debris, making the nerve regeneration easier. Macrophages not only remove axon and myelin debris, but also participate in the production of mitogenic factors for SCs [23,27]. 

With respect to saline, BoNT/A increased the expression of markers related to proliferation of mast cells, of macrophages, and of SCs, which may support an early clearance of fragmented myelin and of its debris [8,24], an important precondition for the axonal regeneration after peripheral nerve injury [28]. In parallel, BoNT/A reduced the expression of P0 and PMP22 myelin peripheral proteins and the presence of the myelin ovoids, which characterize peripheral nerves in CCI-saline mice, indicating an anticipation of myelin phagocytosis processes in CCI mice [24]. 

A different pattern was obtained in CCI-BoNT/B mice (Table 1). With respect to saline, BoNT/B reduced proliferation of SCs (GFAP; but not S100β), did not increase the expression of macrophages, and increased only the expression of mast cells, even if to a lesser extent than BoNT/A. Instead, similarly to BoNT/A, BoNT/B reduced the expression of P0 and PMP22. However, the finding that the functional recovery appears slowed down in CCI-BoNT/B mice with respect to CCI-BoNT/A mice, could indicate that myelin phagocytosis, which *per se* should facilitate the nerve regeneration, is not sufficient if not accompanied by other relevant processes, e.g., the increased proliferation of macrophages and SCs observed in CCI-BoNT/A. In this regard, we should consider that activated mast cells at the periphery release different proinflammatory cytokines that directly stimulate receptors on axons or cell bodies, and may result in neuronal activation. Some released factors, such as chemokines, serve as chemoattractants and activators for other immune cells, such as neutrophils and leukocytes [29], which help the recruitment of hematogenous macrophages at the site of injured nerve [25]. The modest increase of mast cells, not accompanied by an enhancement of macrophages observed in CCI-BoNT/B mice, could indicate a reduced pro-regenerative effect of this serotype. On the other hand, it has to be considered that neurofilaments in BoNT/B appear particularly disorganized and deranged, similarly to saline nerves, while the cytoskeleton of BoNT/A-treated nerves are much more structurally organized. 

Regarding the SC marker S100β, we found a similar expression in all CCI mice groups. This result seems to be in contrast with Cobianchi et al. [30] where BoNT/A-injected regenerating nerves showed a higher expression of S100β with respect to saline-injected regenerating nerves. The discrepancy is only apparent because the higher expression of S100β was detected in distal portion of crushed nerve. In the proximal portion of crushed nerve, as in the present experimental conditions, the expression of S100β in BoNT/A-injected nerves does not differ from saline-injected nerves, also in Cobianchi et al. [30]. 

After damage to the PNS, spinal microglia cell density increases through the migration of these cells from other sites and through local proliferation. In response to traumatic nerve injury, microglia exhibit variable alterations in function and morphology, from a resting state, where the cell body is small with long and thin processes, into an activated state, in which cells present an amoeboid form [31]. Microglial recruitment and activation in spinal cord is accompanied by proliferation and activation of astrocytes. Compared with the microglial response, astrocyte proliferation begins relatively late, progresses slowly, and is sustained for a longer period [32].

By IF analysis, we revealed that, compared to CCI-saline mice, CCI-B7.5 mice displayed no difference in the expression of resting and phosphorylated microglia, both in dorsal and ventral horns. On the other hand, in CCI-B7.5 mice, the toxin exerted a strong effect on the astrocytes of dorsal horn, resulting in lower abundance and activation. Unlike dorsal horn, in ipsilateral ventral horn of CCI-B7.5 mice, astrocytes, even if decreased in number, showed still a strong activation, as indicated by the intense expression of phosphorylated astrocytes and by their morphology. 

Previous studies have already shown that, after ligature of sciatic nerve, glial activation in spinal cord can be reduced by peripheral administration of BoNT/A [13]. In fact, Vacca et al. [33] have shown that treatment with BoNT/A induces significant reduction of activated and not activated glial cells (both microglia and astrocytes) in dorsal and ventral horns of mice. This effect can be explained by indirect but also by direct action of BoNT/A on spinal cord. Marinelli and colleagues [12] showed that BoNT/A, injected into plantar surface of injured paws of CCI-mice, is transported from the peripheral nerve endings to the spinal cord, where it can be transcytosed by nociceptive fibers to astroglial cells. 

Our results showed that, as BoNT/A, also BoNT/B might act at both peripheral and central levels. Previous studies have already suggested possible transsynaptic/transcytotic effects of ipl BoNT/B, which would be transported centrally to inhibit spinal activation and affect nociceptive processes [17,18].

The target of enzymatic activity for BoNT/B, i.e., the protein VAMP2, was found expressed in sensory neurons located in laminae I-VI and X of the spinal cord [34,35]. Analgesic effect of BoNT/B observed in the behavioral tests may be explained by considering its proteolytic activity on VAMP2 protein, which blocks the release of excitatory substances from primary afferents of dorsal horn neurons. Moreover, a contribution to the analgesic effect of BoNT/B may be due to the reduction of astrogliosis at the dorsal horn of spinal cord in CCI-B7.5 mice, as already reported for BoNT/A [33]. In particular, a possible direct action of BoNT/B on dorsal horn astrocytes, probably by transcytosis, could inhibit the glutamate release from astroglial cells and, consequently, contribute to the reduction of pain. Effectively, the possibility that BoNTs can be transcytosed to astrocytes has been already suggested, at least for BoNT/A [23], as it has also been demonstrated, the ability of BoNT/B to inhibit the release of glutamate by astrocyte cultures [36]. 

In conclusion, results of IF experiments associated with behavioral and functional data indicate that BoNT/B exerts analgesic effects on allodynia, similarly to BoNT/A, but does not exert beneficial action on functional recovery of the injured hindlimb, reproducing only some of the effects induced by BoNT/A on structural alterations in CCI-injured nerves. Finally, at spinal cord level, reactive astrogliosis is still present in ventral horns of CCI-B7.5 mice, where terminals of motoneurons are prevalently localized, an effect that could contribute to the impairment of functional recovery. 

Although further research will be needed to clarify and better understand the molecular mechanism underlying the different effects of BoNT/A and BoNT/B in neuropathic pain, data presented in this study show clearly that the two botulinum serotypes are not fully interchangeable. This result is particularly relevant in view of a therapeutic approach aimed at neutralizing the neuropathy induced by peripheral nerve injury; in fact, while both toxins are able to act as analgesics, BoNT/B, differently from BoNT/A, does not induce a parallel acceleration of functional recovery of the injured limb.

## 4. Materials and Methods

### 4.1. Animals

CD1 male mice (Charles River, Como, Italy) were used in the present study. Upon arrival, the animals were housed in groups of four in standard breeding cages (21 × 21 × 12 cm), at constant temperature (22 ± 1 °C), under 12 h light/dark cycle (07:00 a.m.–07:00 p.m.), with food and water ad libitum. At the time of surgery, they were approximately 3 months old and weighed 40–45 g. Experiments were carried out from 10:00 a.m. to 02:00 p.m. Experimenters were blind with regard to which treatment group each subject belonged. All the experiments were conducted in accordance with the Italian National Law (DL 26/2014), with the European Union Council Directive of 22 September 2010 (2010/63/EU), with the NIH guidelines on animal care, and with the ethical guidelines of the Committee for Research and Ethical Issues of IASP [37]. 

### 4.2. Surgical Procedure

Following the procedure originally proposed by Bennett and Xie [38], adapted to mice, CCI of sciatic nerve was used as model of peripheral nerve injury that can evoke neuropathic pain symptoms. Surgery was performed under anesthesia induced by intraperitoneal (ip) injection of a mixture of ketamine (100 mg/kg) and xylazine (5 mg/kg) purchased from Sigma-Aldrich (St. Louis, MO, USA). The CCI was obtained by means of three unilateral ligatures of sciatic nerve. The middle third of the right sciatic nerve was exposed through a 1.5 cm longitudinal skin incision. Three ligatures (7-0 prolene, Ethicon) were tied loosely around the nerve. The wound was then closed with silk suture (4-0 vicryl, Ethicon) and the mouse was allowed to recover in a heated cage until all reflexes were normalized. The injured and uninjured hindpaws were named as ipsilateral and contralateral hindpaws, respectively.

### 4.3. Pharmacological Treatments and Experimental Groups

Isolated and purified 150 kDa di-chain BoNT/A and /B were a kind gift from Prof. C. Montecucco and Prof. O. Rossetto (University of Padova). The toxins were frozen in liquid nitrogen and stored at −80 °C in 10 mM NaHEPES, 150 mM NaCl, pH 7.2. Stock solutions were tested for activity in the ex vivo mouse hemidiaphragm model, and in the in vitro cleavage of SNAP-25 for BoNT/A and VAMP/synaptobrevin for BoNT/B, respectively. Injectable solutions of BoNTs were freshly made by dilution in saline (0.9% NaCl). 

After verification of the onset of neuropathy at day 3 post CCI (D3), CCI mice were randomly assigned to different groups according to their experimental use. At D5, selected solutions of BoNTs (BoNT/B = 5 or 7.5 pg/mouse, indicated as B5 or B7.5, respectively; BoNT/A = 15 pg/mouse, indicated as A15) or saline (0.9% NaCl) were ipl injected into plantar surface of the injured hindpaw. Doses of BoNT/A and /B were chosen on the basis of neurotoxicity (LD50: 0.5–1.0 × 10^−6^ mg/kg) and previous studies [11,19,39,40]. These studies showed that BoNT/B is more toxic than BoNT/A (see also [41]), therefore, doses of BoNT/B higher than 7.5 pg/paw were not tested. Behavioral investigations were carried out in CCI-saline, CCI-B5, and CCI-B7.5 mice (*n* = 10 mice/group). Immunofluorescence (IF) analysis was carried out on sciatic nerve of naïve, CCI-saline, CCI-B7.5 and, for comparison, on nerves of CCI-A15 mice (*n* = 3 mice/group), and on spinal cord of CCI-saline and CCI-B7.5 mice (*n* = 3 mice/group).

### 4.4. Behavioral Tests

#### 4.4.1. Measurement of Mechanical Nociceptive Threshold

The onset of CCI-induced neuropathy was assessed by measuring the threshold of both hindpaws to normally non-noxious punctuate mechanical stimuli. The hindpaw nociceptive threshold, measured by an automatic von Frey apparatus (Dynamic Plantar Aesthesiometer, Ugo Basile, Italy), was expressed as the force (in grams) at which mice withdrew their paws in response to the mechanical stimulus. For habituation, mice were placed in plastic cages with a wire net floor 5 min before the experiment. The mechanical stimulus was applied to the mid-plantar surface of the hindpaw to induce a slight pressure to the skin. At each testing day, ipsi- and contralateral withdrawal thresholds were taken as mean of three consecutive measurements per paw with 10 s interval between each measurement. Mice were tested each day from D3 to D7, every two days from D10 to D21, and every ten days from D31 to D101.

#### 4.4.2. Measurement of Weight Bearing

Weight bearing on the two hindlimbs was determined by incapacitance test (Linton Instrumentation, Norfolk, UK). The apparatus used was adapted for mice with a strain gauge/amplifier resolution of 0.03 g, and a strain gauge/amplifier accuracy of 0.1 g. Mice were carefully placed in an angled Plexiglas chamber positioned with hindpaws on the two separate force plates. Care was taken to ensure that the animal weight was directed onto the force plates, and not dissipated through the walls of the chamber. The force exerted by each hindlimb (measured in grams) was automatically averaged over a 5 s period and, for each animal, measurement was repeated three times with 5 min interval. Mice were tested the same day of the measurement of mechanical nociceptive, with weight bearing performed before and with 1 h of rest between the two tests. The weight bearing was calculated by the equation

[ipsilateral weight/(ipsilateral weight + contralateral weight)] × 100

#### 4.4.3. Walking Footprint Analysis and Sciatic Static Index

The gradual recovery of the paw functionality was monitored by analyzing individual free-walking patterns and by measuring several footprint parameters to calculate the sciatic static index (SSI). Footprints were recorded by dipping in black ink the hindpaws of mice and leaving them to freely walk along a Perspex runaway corridor (15 × 5 × 50 cm) lined with white paper. Footprint parameters were calculated from at least five footprints recorded on three different walking track runaways. Mice were tested at D11, D19, and every ten days from D32 to D102. SSI was evaluated using the equation proposed by Baptista et al. [42]:

SSI = +101.3 × (ITS-CTS)/CTS − 54.03 × (IPL-CPL)/CPL − 9.5

considering ITS and CTS as the toe spreads, i.e., the distance between 1st to 5th digit, of the ipsilateral and contralateral hindpaw, and IPL and CPL as the paw length, i.e., the distance between the tip of the third toe and the most posterior aspect of the ipsilateral and contralateral hindpaw. Based on this equation, a value of SSI close to 0 corresponds to normal function, while a value close to −100 is equivalent to complete functional loss. 

### 4.5. Immunohistochemistry Assays

#### 4.5.1. Immunostaining of Sciatic Nerve

At D7, mice assigned to sciatic nerve immunostaining were anaesthetized with a mixture of ketamine (100 mg/kg, ip) and xylazine (5 mg/kg, ip) purchased from Sigma-Aldrich (St. Louis, MO, USA) and the lesioned part of the ipsilateral sciatic nerve, including ligatures, were removed immediately before perfusion and kept in immersion for 48 h in paraformaldehyde (PFA; Sigma-Aldrich, Milan, Italy) 4% in 0.1 M phosphate-buffer saline (PBS; Sigma-Aldrich, Milan, Italy), pH 7.2, room temperature. Nerves were cryoprotected with a solution of 30% (w/v) sucrose in PBS, then stored at −80 °C until sectioning.

Longitudinal sections (25 μm thick) of sciatic nerves segment, including the portion with ligature, were cut on cryostat microtome and mounted directly on slides. For IF staining, sciatic nerves sections were washed three times in PBS, and then incubated overnight in Triton (0.3% in PBS; Sigma-Aldrich) with the following primary antibodies in different combinations: mouse anti-GFAP (monoclonal; 1:100; Sigma-Aldrich); rabbit anti-PMP22 (polyclonal; 1:100; Sigma-Aldrich); chicken anti-P0 (polyclonal; 1:100; Millipore, Vimodrone, Italy); rabbit anti-NF200 (polyclonal; 1:100; Sigma-Aldrich); mouse anti-S100β (polyclonal; 1:100; Sigma-Aldrich); rat anti-CD11b (monoclonal; 1:100; Bio-Rad, Segrate, Italy); mouse anti-CC1 (monoclonal; 1:100; Santa Cruz, Segrate, Italy). Afterwards, sections were washed three times with PBS, and incubated for 2 h at room temperature, in Triton with one of the following secondary antibodies: donkey anti-mouse fluorescein-conjugated (FITC; 1:100; Jackson ImmunoResearch, West Grove, PA, USA); goat anti-rabbit fluorescein-conjugated (FITC; 1:100; Santa Cruz); goat anti-rabbit rhodamine-conjugated (TRITC; 1:100; Jackson ImmunoResearch); donkey anti-chicken rhodamine-conjugated (TRITC; 1:100; Jackson ImmunoResearch). Sections were washed three times in PBS, incubated in PBS with the nuclear marker Hoechst 33258 (1:1000; Sigma-Aldrich) for 5 min, washed again three times in PBS, mounted with glycerol/PBS 3:1 mounting medium, and cover slipped.

#### 4.5.2. Immunostaining of Spinal Cord

At D7, mice assigned for spinal cord immunostaining were anaesthetized with chloral hydrate (500 mg/kg ip; Sigma-Aldrich, Italy) and transcardiacally perfused with saline (100 mL) followed by PFA (100 mL). After perfusion, the entire spinal cord of each animal was removed and kept in PFA for 24 h. Spinal cords were cryoprotected with a solution of 30% (w/v) sucrose in PBS, then stored at −80 °C until sectioning.

Transverse sections (40 μm thick) of L4/L5 spinal cord segment were cut on a cryostat microtome and collected in PBS for free-floating double IF procedures. Ipsi- and contralateral side of sections were recognized by marking the spinal cord with a notch on the contralateral side before mounting spinal cord trunk on the chuck of the cryostat. Sections were first incubated overnight in Triton with different combinations of the following primary antibodies: mouse anti-GFAP (monoclonal; 1:100; Sigma-Aldrich); rat anti-CD11b (monoclonal; 1:100; Bio-Rad); rabbit anti-p-p38 (polyclonal; 1:100; Santa Cruz). Afterward, sections were washed three times in PBS, and incubated for 2 h at room temperature in Triton, with one of the following secondary antibodies: donkey anti-mouse or anti-rat fluorescein-conjugated (FITC; 1:100; Jackson ImmunoResearch); goat anti-rabbit rhodamine-conjugated (TRITC; 1:100; Jackson ImmunoResearch). Sections were washed three times in PBS, incubated in PBS with the nuclear marker Hoechst 33258 (DAPI; 1:1000; Sigma-Aldrich) for 5 min, washed again three times in PBS, mounted on slides with glycerol/PBS 3:1, and cover slipped.

#### 4.5.3. Confocal IF Analysis

Low (10×) magnification images of spinal cord sections, and high (63×) magnification images of spinal cord and sciatic nerve sections, were captured by laser scanning confocal microscopy (TCS SP5 microscope, Leica Microsystem, Buccinasco, Italy) connected to digital camera diagnostic instruments operated by I.A.S. software (Delta Systems, Bergamo, Italy).

In sciatic nerve sections, only the part of nerve including the ligature was used. The analysis of IF staining (in term of emitted fluorescence) was performed by using the ImageJ software (version 1.41, National Institutes of Health, Bethesda, MA, USA). The fluorescence was quantified with RGB (red, green, blue) method, which uses brightness values for calculation [43]. The IF images were analyzed in three mice, considering three nerve sections per mouse.

In spinal cord sections, to quantify glial cells immunoreactivity (IR), IF images of the ipsilateral side of the dorsal horn (medial portion of laminae I–IV) and ventral horn (dorsolateral column, lamina IX) from three spinal cord sections per mouse were examined. Quantification was performed using the ImageJ software. Image contrast was adjusted such that the background level just disappeared, and the cell bodies and processes appeared without boundaries; the same cutoff level was used for all images. To quantify GFAP and CD11b IR-cells, and their colocalization with p-p38, the number of positive cells was counted in three mice, considering three spinal cord sections per mouse.

### 4.6. Data Analysis

Data are expressed as mean ± SEM. Statistical analysis of results from behavioral analysis was performed by two-way ANOVA for repeated measure, followed by Tukey–Kramer post hoc comparison. Statistical analysis of results from RGB pixels analysis of IF were performed by one-way ANOVA followed by Fisher PLSD post hoc comparison.

## Figures and Tables

**Table 1:** Summary of differences in the expression of biological markers associated to peripheral nerve injury in CCI-A15 and CCI-B7.5 vs. CCI-saline mice.

Target	Marker	CCI-A15	CCI-B7.5
Non-myelinating SCs	GFAP	↑	↓
Myelinating SCs	S100b	=	=
Peripheral myelin	P0	↓	↓↓
Peripheral myelin	PMP22	↓↓↓	↓↓
Mast cells	CC1	↑↑↑	↑
Macrophages	CD11b	↑↑↑ ^1^	=

^1^ Upward arrows, downward arrows, or equal sign indicate, respectively, enhanced, reduced, or equal expression of corresponding markers in CCI-BoNTs vs. CCI-saline mice. One, two, or three arrows indicate significant difference: *p* < 0.05 (↑), *p* < 0.01 (↑↑), or *p* < 0.001 (↑↑↑).
